# The role of metabolic reprogramming in pancreatic cancer chemoresistance

**DOI:** 10.3389/fphar.2022.1108776

**Published:** 2023-01-09

**Authors:** Chang Liu, Changfeng Li, Yuanda Liu

**Affiliations:** Department of Endoscopy Center, China-Japan Union Hospital of Jilin University, Changchun, China

**Keywords:** metabolic reprogramming, pancreatic cancer, chemoresistance, glycolysis, glutamine metabolism, fatty acid synthesis

## Abstract

Pancreatic cancer is characterized by hidden onset, high malignancy, and early metastasis. Although a few cases meet the surgical indications, chemotherapy remains the primary treatment, and the resulting chemoresistance has become an urgent clinical problem that needs to be solved. In recent years, the importance of metabolic reprogramming as one of the hallmarks of cancers in tumorigenesis has been validated. Metabolic reprogramming involves glucose, lipid, and amino acid metabolism and interacts with oncogenes to affect the expression of key enzymes and signaling pathways, modifying the tumor microenvironment and contributing to the occurrence of drug tolerance. Meanwhile, the mitochondria are hubs of the three major nutrients and energy metabolisms, which are also involved in the development of drug resistance. In this review, we summarized the characteristic changes in metabolism during the progression of pancreatic cancer and their impact on chemoresistance, outlined the role of the mitochondria, and summarized current studies on metabolic inhibitors.

## 1 Introduction

Pancreatic cancer (PC) is a highly aggressive malignant tumor, mainly pancreatic ductal adenocarcinoma, which is the seventh leading cause of cancer death worldwide (Sung et al., 2021). Due to the insidious onset of PC, the lack of effective early screening tools, and the early initiation of metastasis, many patients are diagnosed at an advanced stage and are unable to obtain radical resection. Thus, the treatment is still mainly based on chemotherapy. Gemcitabine (GEM), also known as dFdC, a deoxycytidine analogue being phosphorylated intracellularly after ingestion (Mini et al., 2006), inhibits ribonucleotide reductase activity and incorporates into DNA to impede tumor cell proliferation. 5-Fluorouracil (5-Fu) is a thymidylate synthase (TS) inhibitor that interferes with tumor cell DNA synthesis and incorporates metabolites into RNA to impair protein synthesis (Longley et al., 2003). The chemotherapy for PC is still dominated by regimens based on the two drugs above, and the current first-line chemotherapy protocol is fluorouracil, leucovorin, irinotecan, and oxaliplatin (FOLFIRINOX), or gemcitabine (GEM) combined with the nab-paclitaxel (Wood et al., 2022). However, PC has an overall limited response to chemotherapy and is prone to drug tolerance, with a 5-year survival rate of only approximately 10%, making it essential to overcome chemoresistance (Rawla et al., 2019).

Chemotherapy resistance in PC can be caused by a combination of multiple factors, including altered expression levels of drug targets and drug metabolizing enzymes, activation of intracellular survival signaling pathways and inhibition of apoptosis, interactions between PC cells and the tumor microenvironment (TME) such as mesenchymal and pancreatic stellate cells (PSCs), epithelial mesenchymal transition, immune escape of cancer stem cells, epigenetic alterations and metabolic abnormalities, and changes in microRNA expression levels (Cao et al., 2015; Zheng et al., 2015; Ireland et al., 2016; Dauer et al., 2017). With the deeper studies on tumor metabolism in recent years, metabolic alterations in cancer cells are also considered to be one of the non-negligible reasons for the occurrence of chemoresistance (Fujimura et al., 2014). For example, an increase in the M2 isoform of pyruvate kinase (PK), a key enzyme of glycolysis in PC, causes GEM resistance (Calabretta et al., 2016). Inhibition of fatty acid synthase (FASN), which is increased in lipid metabolism, affects PC cell stemness and induces endoplasmic reticulum stress, thus increasing the sensitivity to GEM(Tadros et al., 2017).

Metabolic reprogramming is a hallmark of malignant tumors, including the reprogramming of glucose, lipid, and amino acid metabolism, which occurs throughout PC development and evolves into different metabolic phenotypes according to the needs of different stages. Understanding the corresponding new metabolic phenotypes in the context of drug resistance can provide ideas for targeting tumor metabolism to improve chemotherapy sensitivity. Mitochondria are pivotal points among the regulation of metabolism in the three nutrient substances (Faubert et al., 2020).

In this review, we focused on the molecular mechanisms and latest advances of metabolic reprogramming affecting PC chemoresistance, considered the role of the mitochondria, and suggested new approaches to overcoming chemoresistance.

## 2 Alterations in glucose metabolism

The Warburg effect (aerobic glycolysis) is an integral part of metabolic reprogramming in PC, with the reverse Warburg effect, oxidative phosphorylation pathway (OXPHOS), pentose phosphate pathway (PPP), and hexosamine biosynthetic pathway (HBP) as adjuncts, which work together to enable cancer cells to adapt to varying environments and influence chemotherapeutic efficacy by modifying metabolic pathways.

### 2.1 Glycolysis

The dense tumor stroma of PC leads to vascular hypoperfusion, which causes hypoxic conditions (DuFort et al., 2016). Tumor cells consume a lot of energy for rapid proliferation, favoring the provision of energy through better and efficient glycolytic pathways. At present, it is believed that the Warburg effect does not arise from mitochondrial dysfunction in tumor cells, but from the synergy of overexpression of hypoxia-inducible factor-1 (HIF-1), activation of oncogenes (KRAS, c-MYC), loss of tumor suppressors (mutant P53, PTEN, microRNAs), activation of signaling pathways, the TME, and epigenetic modifications (Vaupel and Multhoff, 2021). This drives the overexpression of glucose transporter proteins, key enzymes of glycolysis, activates drug resistance-related signaling pathways, enhances glycolytic flux, and promotes PC development while enhancing tolerance to chemotherapy. The mechanisms by which glycolysis affects chemoresistance are described below in terms of the interactions between key enzymes and transporters of the glycolytic process, signaling pathways, metabolites, and the TME.

#### 2.1.1 Changes in key enzymes and transporters in glycolysis

Glucose transporters (GLUTs) are responsible for transporting glucose across the cell membrane into the cytoplasm. Both GLUT1 and *SLC2A1*, which encode GLUT-1 protein, are highly expressed in PC cells and are associated with poor clinical prognosis (Yu et al., 2017; Kurahara et al., 2018; Du et al., 2021). Activation of KRAS, c-MYC, and HIF-1α in tumors activates GLUT1, which is involved in chemoresistance by promoting the overexpression of GLUT-1 to activate NF-κB and mTOR targets. Sodium-glucose co-transporter (SGLT) is a second type of glucose transporter that is different from GLUT, encoded by the *SLC5* gene family. Canagliflozin (CanA) is an inhibitor of SGLT2, which was shown to increase the cytotoxicity of GEM by downregulating GLUT-1 and LDHA levels in recent study (Xu D. et al., 2020).

Hexokinase (HK), the first key enzyme of glycolysis, converts glucose into glucose-6-phosphate. In four isozymes, HK2 is highly expressed in PC and associated with shorter overall survival (OS) (Krasnov et al., 2013; Yu et al., 2019a). HK2 binds to the mitochondria *via* voltage-dependent anion channels (VDACs), which inhibits or closes mitochondrial permeability transition pores. Through inhibiting the release of cytochrome c and other apoptotic factors, the apoptosis is suppressed, and chemoresistance is promoted (Ma et al., 2019). Reactive oxygen species (ROS) generated by GEM can promote HK2 dimerization and binding to VDAC (Fan et al., 2019). The phosphatidylinositol-3-kinase (PI3K)/protein kinase B (Akt)/mammalian target of rapamycin (mTOR) pathway also enhances the binding of HK2 to the mitochondrial membrane to induce drug resistance. Upregulation of HK2 further increases glycolytic flux, raises ATP levels, and interacts with HIF-1α to trigger chemoresistance. 2-deoxy-D-glucose (2-DG), a nonmetabolizable glucose analogue, is an inhibitor of HK. 2-DG in combination with either GEM or oxaliplatin improves the chemosensitivity of PC cells (Dai et al., 2020). Ikanomycin (IKA) also blocks glycolysis by targeting HK2, reduces tumor volume in mice with PC xenografts, and enhances the chemotherapeutic response of PC cells to GEM (Jiang et al., 2020).

Phosphofructokinase (PFK), the second key enzyme of glycolysis, converts fructose-6-phosphate (F-6-P) to glucose-1,6-bisphosphate. PFK has two isoforms; PFK1 catalyzes the above rate-limiting step, and PFK2 mediates the conversion of F-6-P to glucose-2,6-bisphosphate (F-2,6-BP), and F-2,6-BP is the strongest variable activator of PFK1. PFK2, also known as 6-phosphofructo-2-kinase/fructose-2,6-bisphosphatase (PFKFB), has four isoforms, of which PFKFB3 is a downstream target of HIF-1α that is significantly elevated in PC and correlates with poor clinical prognosis (Shi et al., 2017; Wang F. et al., 2020). Recently, it was found that PFKFB2 is also overexpressed in pancreatic cancer, and mRNA spliceosomes of different PFKFB2 are localized in the cytoplasm and nucleus, respectively, affecting F-2,6-BP levels, glycolytic activity, and cell proliferation (Ozcan et al., 2020). EGCG, a polyphenol in green tea, impacts glycolysis by reducing PFK and pyruvate kinase (PK) activity to inhibit extracellular acidification. EGCG also shows significant sensibilization on GEM in PC cells and PC xenografts (Wei et al., 2019).

PK, the last key enzyme of glycolysis, converts phosphoenolpyruvate to pyruvate. PKM2 is the main isoform in tumors, and the conversion of PKM1 to PKM2 is thought to be a marker for switching to aerobic glycolysis and promoting tumorigenesis. PKM2 expressions are elevated in PC patients and are associated with a worse prognosis (Bandara et al., 2018). PKM2 acts in both the cytoplasm and nucleus to induce chemoresistance. In the cytoplasm, it facilitates glycolysis, while in nucleus, it is phosphorylated and serves as a protein kinase to regulate gene expression. Upregulation of polypyrimidine binding protein (PTBP1) in PC promotes the production of PKM2 isoform, which in turn leads to GEM resistance (Calabretta et al., 2016). PKM2 also causes P53 inactivation by inhibiting P38-mitogen-activated protein kinase (MAPK), leading to GEM resistance, and knockdown of PKM-2 dramatically enhances GEM-induced apoptosis in PC (Kim et al., 2015).

Lactate dehydrogenase (LDH) catalyzes pyruvate into lactate. LDHA is overexpressed in PC and is an independent predictor of poor OS in PC patients (Jiang et al., 2021b). LDHA also acts as a direct target of HIF-1α and c-MYC, contributing to biosynthesis and glycolysis to ensure fuel supply for energy and anabolism in cancer cells. Besides, LDHA promotes the expression of anti-apoptotic proteins and reduces ROS production, thereby preventing apoptosis and eliciting chemoresistance (Comandatore et al., 2022). Mitosis-related gene *Fam83D* expression is augmented in PC, and knockdown of *Fam83D* decreases c-MYC and LDHA expression *via* the Wnt/β-catenin signaling pathway, resulting in increased PC cell sensitivity to GEM (Hua et al., 2021). NHI is an LDHA inhibitor that functions synergistically with GEM to raise GEM-required dCK expression. Thus, they increase GEM-containing DNA synthesis, affect the cell cycle and induce apoptosis, and substantially restore PC cell sensitivity to GEM (Maftouh et al., 2014). NHI-Glc-2, a novel inhibitor, dual-targets LDHA and GLUT-1 and makes anti-cancer effects more pronounced in combination with GEM (El Hassouni et al., 2020).

Glyceraldehyde-3-phosphate dehydrogenase (GAPDH) catalyzes 3-phosphoglyceraldehyde dehydrogenase into 1,3-diphosphoglycerate and NADH, whose mRNA and protein levels are elevated in PC. Iodoacetate, a GAPDH inhibitor, and 3-Bromo-Isoxazoline derivatives have both shown antiproliferative effects on PC cells (Bhardwaj et al., 2010; Pacchiana et al., 2022). Mutant P53 arrests nuclear translocation of GAPDH by activating AKT signaling and repressing AMPK signaling, which stabilizes GAPDH in the cytoplasm, and strengthens glycolysis in cancer cells while restraining nuclear GAPDH-mediated cell death mechanisms. Disrupting GAPDH fixation in the cytoplasm restores the sensitivity of PC cells to GEM, suggesting a potential personalized therapeutic option for patients carrying the mutant P53 gene (Butera et al., 2018).

Enolase (ENO), which catalyzes the formation of 2-phosphoglycerate into phosphoenolpyruvate, has five isoforms. In PC cells, high ENO-1 levels contribute to tumor migration and invasion by inducing the activation of fibrinogen into fibrinolytic enzymes, which degrade the dense ECM. In PC patients, the higher the level of ENO1 expression, the shorter the OS (Wang C. et al., 2022). The increased level of ENO-1 expression in hypoxia is associated with the expression of HIF-1α (Sun et al., 2017). Silencing ENO1 enhances the sensitivity of PC cells to GEM by modulating redox, which may be related to the regulation of cell proliferation, apoptosis, and the cell cycle by increased intracellular ROS (Wang L. et al., 2019). Recently developed ENO-1 DNA vaccination in combination with treatments such as ENO-1 inhibitors and chemotherapy may also enhance the therapeutic effect of PC (Cappello et al., 2018).

Monocarboxylic acid transporters (MCTs), of which MCT1 and MCT4 are mainly involved in lactate transport, are highly expressed in PC subtypes with active glucose metabolism and are associated with high recurrence rates. PC cells strongly express MCT1 and MCT4 to accelerate lactate efflux, thereby avoiding the accumulation of excess lactate produced by aerobic glycolysis that causes cell death (Kong et al., 2016). MCT1 transports lactate in both directions. MCT4 mainly promotes lactate efflux from cells. Inhibition of MCT4 with siRNA increases the sensitivity of PC cells to GEM (Lee et al., 2021). Meanwhile, MCT1-driven lactate input is a critical process for the reverse Warburg effect. PC cells expressing MCT1 (e.g., BxPc3) are protected from GEM hypoxia-induced apoptosis by lactate. Administration of the MCT1 inhibitor 7ACC2 or knockdown MCT1 effectively reduces GEM resistance in MCT1-positive PC cells, providing a target for PDAC cells dependent on the reverse Warburg effect of MCT1 (Sandforth et al., 2020). CPI-613 is a potent inhibitor of two enzymes, pyruvate dehydrogenase (PDH) and alpha-KGDH, in the tricarboxylic acid (TCA) cycle and has shown significant inhibition of PC proliferation in combination with the LDH inhibitor galloflavin or the MCT inhibitor alpha-cyano-4-hydroxycinnamic acid (Kumstel et al., 2022). The efficacy of CPI in collaboration with FOLFIRINOX has also been shown to be efficacious in phase 1 clinical studies in metastatic PC patients, with overall objective response rate and overall median survival at the maximum dose being superior to FOLFIRINOX alone (Alistar et al., 2017).

#### 2.1.2 Glycolysis and signaling pathways

As the main gluconeogenic pathway in PC cells, Warburg effect is characterized by enhanced glucose uptake and conversion to lactate. The Warburg effect is achieved by the interaction and upregulation of key factor HIF-1α expression, mutation of proto-oncogenes/oncogenes, and activation of multiple signaling pathways. Concurrently, key glycolytic enzymes and transporters downstream of the signaling pathway can also serve to activate signaling pathways associated with chemoresistance through their own or other protein mediators. The complementary synergy between the key links of glycolysis and oncogene signaling pathways provides the possibility for PC cells to tolerate chemotherapy.

HIF-1α, a nuclear protein with increased secretion under hypoxia, is readily degraded under normoxia and stable under hypoxia. It induces the expression of almost all genes encoding glucose transporter proteins and key enzymes of glycolysis and directs the metabolic conversion of tumors from oxidative phosphorylation (OXPHOS) to aerobic glycolysis. It is also a central regulator of PC cells to bring the Warburg effect. Oncogene signaling pathways such as PI3K/AKT and MAPK/extracellular signal-regulated kinase (ERK) are upstream signals for HIF-1α. After entering the nucleus, increased HIF-1α acts as a transcription factor and binds to corresponding target sequence. Then, the enhanced glycolysis by promoting the expression of glycolytic key enzymes and transporter proteins (such as GLUT1, HK2, PKM2, and LDHA) and the inhibited mitochondrial respiration with reduced ROS accumulation reduce chemotherapy sensitivity. hENT1 is a nucleoside transporter required for GEM to enter cells. It restores the sensitivity of PC cells to GEM by suppressing HIF-1α-mediated glycolysis (Xi et al., 2020). Inhibition of HIF-1α mediated glycolysis restores PC cells’ sensitivity to GEM chemotherapy. Prolyl 4-hydroxylase subunit alpha 1 (P4HA1) has a positive feedback loop with HIF-1α. Silencing P4HA1 gene significantly improved drug resistance in PC cells (Cao et al., 2019). Mucin 1 (MUC1) is an oncogenic mucin that increases the stability of HIF-1α and reduces the sensitivity of PC cells to GEM and 5-Fu by regulating multiple drug resistance (MDR) gene expression through the AKT pathway (Tréhoux et al., 2015). The GEM resistance-associated gene stanniocalcin-1 (STC1) is related to the prognosis of patients with adjuvant GEM chemotherapy after surgery. Recently, cell and animal models have further validated that STC1 mediates chemoresistance to PC under the action of the HIF-1α/STC1/PI3K-AKT axis (Zhao et al., 2022). Long noncoding RNA antisense RNA1 of HIF1α (lncRNA HIF-1α-AS1) also promotes GEM resistance in PC cells through the AKT/YB1/HIF-1α pathway (Xu F. et al., 2021). Digoxin, a HIF-1α inhibitor, has shown reversal of GEM resistance in both cellular and animal studies (Shukla et al., 2017). HIF-1α is an essential link in the development of chemoresistance in PC due to metabolic reprogramming and a potential therapeutic target.

The downstream target of PI3K/Akt is mTOR, while the downstream transcription factors of mTOR include star molecules such as HIF-1α, c-MYC, and forkhead box O(FOXO), which are involved in various aspects of hypoxia adaptation and cell survival pathways. Under the regulation of this pathway, they help in forming the drug resistance phenotype of cancer cells. AKT activation increases the activity and expression of GLUT1 and HK2, switching glucose metabolism from oxidative phosphorylation to aerobic glycolysis (Liu et al., 2020). While maintaining the dynamic balance of glycolysis, the AKT/mTOR signaling pathway induces drug-resistant cells to overexpress c-MYC, which directly stimulates glucose uptake and enhances glycolysis (Ma and Zong, 2020). mTOR targets the Warburg effect. Administration with mTOR inhibitor everolimus (Evr) suppresses aerobic glycolysis by decreasing glucose, lactate, and ATP levels and inhibiting the expression of GLUT1, LDHB, HK2, and PKM2. Evr also upregulates pro-apoptotic proteins Bax and cyt-c and downregulates anti-apoptotic protein Bcl2 to foster apoptosis. Combined with GEM in nude mice PC xenograft models, it significantly reduces the size of GEM-resistant tumors (Cui et al., 2021). Berberine and 4-AAQB also overcome GEM resistance in PC by modulating the Rap1/PI3K/Akt and PI3K/Akt/MDR1 signaling pathways, respectively (Chen Y. Y. et al., 2022; Okuno et al., 2022).

PC is associated with multiple gene mutations, the most common of which are KRAS and TP53, resulting in more than 90% and 50% of cases respectively (Cao et al., 2020). KRAS is a RAS protein that participates in the RAS/RAF/MAP-ERK kinase (MEK)/ERK signaling pathway, which upregulates a variety of key glycolytic enzymes (such as GLUT1, HK1/2, phosphofructokinase, LDHA) and HIF-1α, stimulating glucose uptake and promoting glycolysis. KRAS also reduces ROS production and related apoptosis by transporting glucose to anabolic pathways such as the pentose phosphate pathway (PPP) (Grasso et al., 2017). The tumor suppressor gene *FBW7* is degraded after the KRAS mutation activates ERK. Overexpression of FBW7 can improve the GEM sensitivity of PC by upregulating ENT1 (Hu et al., 2017). The combination of SRC family kinase (SFK) inhibitor dasatinib and MEK inhibitor trametinib synergistically inhibited PC cell colony formation and suppressed the growth of MiaPaCa-2 cells transplanted in the lateral abdomen of nude mice (Sinnet-Smith et al., 2022). KRAS mutant PC cells also upregulate carbonic anhydrase 9 (CA9) expression by stabilizing HIF-1α and HIF-2α. Both knocking down CA9 or administration of trametinib reduced GEM-induced glycolysis and increased the sensitivity of PC cells and mouse xenograft tumors to GEM(McDonald et al., 2019). Moreover, fructose-1,6-bisphosphatase (FBP1), a key enzyme of gluconeogenesis, also suppresses GEM-activated ERK by inhibiting the IQ motif containing the GTPase-activating protein 1(IQGAP1)/ERK1/2 signaling pathway, thereby enhancing the anticancer effects of the drug and overcoming drug resistance. However, whether FBP1 antagonizes the Warburg effect by blocking the glycolytic pathway or inhibits the above pathway through non-enzymatic activity to ultimately reverse drug resistance remains to be further investigated (Jin et al., 2017). P53, the gene product of TP53, mediates increased glycolysis by TP53-induced glycolysis and apoptosis regulator (TIGAR). Introduction of WT-TP53 improves the sensitivity of PC cells to multiple chemotherapeutic agents, including EGFR/Ras/Raf/MEK and PI3K/mTORC1/GSK-3 pathway inhibitors, and affects crucial metabolic properties of the cells (McCubrey et al., 2022).

#### 2.1.3 Glycolysis and the TME

The TME is an internal environment composed of tumor cells, immune cells, mesenchymal cells, and active factors, and plays a key role in tumor progression. PC microenvironment is marked by dense mesenchyme, vascular constriction, and immune suppression, resulting in a hypoxic and nutrient-deprived environment for tumor cells, also not conducive to drug transport. Enhanced aerobic glycolysis of PC cells produces large amounts of lactic acid, causing environment acidification. They also interact with PSCs to regulate energy utilization and cause chemoresistance.

Choosing aerobic glycolysis as the main approach to energy production inevitably leads to a large accumulation of lactate, which causes a huge change in pH and is a determinant of acidosis in PC tissues and TME, enhancing tumor invasion and migration (Comandatore et al., 2022). In general, acidosis induces apoptosis and autophagy in normal cells. However, under the effect of mutations in genes such as *KRAS* and *TP53* and adaptive regulation of lactate transport metabolism, PC cells are able to survive in this hypoxic acidic microenvironment and insidiously squeeze the anabolic substrate and survival space of normal cells. Hypoxia selects glycolytic cells to induce acidosis, and acidosis can further select cells with upregulated levels of glycolysis and acid tolerance, thus screening out cancer cells for further proliferation (Nunes, 2020).

Lactate produced by hypoxic cancer cells in the center of tumor is usually rapidly exported extracellularly *via* MCT and then taken up by normoxic cancer cells in the tumor periphery for oxidative metabolism *via* MCT1. This cuts down the glucose consumption of hypoxic cancer cells (Grasso et al., 2017). Lactate transport in tumors *via* MCT is also an important component of the reverse Warburg effect. The reverse Warburg effect is a new idea to explain the shortage of aerobic glycolytic energy supply in tumor cells. It suggests that cancer-associated fibroblast (CAF), a key member of the TME, undergoes aerobic glycolysis to produce energy-rich anabolic products such as lactate and pyruvate. Then, these fuels are transferred to cancer cells *via* MCT to produce large amounts of ATP in the mitochondria, so that PC cells have sufficient energy to continue proliferating and diffuse ROS produced by the mitochondria to the extracellular environment. This is a more efficient way of energy production than aerobic glycolysis in tumor cells alone (Fu et al., 2017; Liang et al., 2022).

Acidification of the TME also contributes to chemoresistance in tumor cells. Rapid and continuous excretion of lactic acid by cancer cells maintains a specific pH gradient of high extracellular acidity and high intracellular alkalinity. Most chemotherapeutic drugs are either weakly acidic or alkalescent. Paclitaxel (alkalescent) is directly neutralized by extracellular acidosis, preventing it from crossing the cell membrane. Weakly acidic drugs are also less active in environments with high intracellular bases (da Silva et al., 2018). Accumulation of lactate and low pH activate the unfolded protein response and PI3K/AKT/mTOR signaling pathway as well, inducing drug resistance. To ensure effective drug delivery to tumor cells in acidic TME, combined therapies targeting CA are being investigated, such as SLC-0111 and GEM for CAIX-positive PC (NCT03450018) (Roma-Rodrigues et al., 2019). Discoidin domain receptor 1 (DDR1) increases drug resistance by binding to collagen in the TME. DDR1 inhibitors in combination with GEM significantly reduce TME matrix components and mitochondrial membrane potential, thereby arresting tumor growth in PC transplanted models and helping to overcome GEM resistance (Ko et al., 2022).

CAF is the main constituent of stromal cells, both promoting fibroproliferation, disturbing drug transport, and providing metabolites involved in the reverse Warburg effect, as previously described. Meanwhile, exosomes (TCA cycle intermediates, amino acids, and lipids) released by CAF enrich the microenvironment. They are capable of inhibiting mitochondrial respiration and increase glycolysis KRAS-independently after uptake by PC cells (Zhao et al., 2016). A study also shows that GEM key inactivating enzymes are less expressed in CAFs. CAFs wrap GEM in cells and prevent drugs from reaching cancer cells, inducing GEM chemotherapy resistance in PCs (Qin et al., 2020).

PSC is an integral part of the TME. In normal pancreatic tissue, it is quiescent and only produces a little ECM. In PC, it is activated by cancer cell signaling and exhibits potent CAF activity to secrete large amounts of ECM. PSCs and PC cells form a complex regulatory network that promotes cancer cell growth, metastasis, and drug resistance. CXCL12 secreted by PSC activates the CXCR4/PI3K/AKT/mTOR pathway, secreted hepatocyte growth factor activates the c-Met/PI3K/Akt pathway, and both factors enhance glycolysis and induce GEM resistance (Xu J. et al., 2020; Lu et al., 2022). Treatments aimed at PSC have been shown to be effective in overcoming chemoresistance. Targeting vitamin D receptors on PSCs helps PSCs to revert to a quiescent state and improves sensitivity to GEM (Sherman et al., 2014). ProAgio targeting integrin αvβ3, highly expressed on PSCs, specifically induces PSC apoptosis, reduces vascular compression by ECM, improves drug transport, and decreases the level of the GEM metabolic enzyme cytidine deaminase, thus improving chemotherapy efficacy (Turaga et al., 2021). Treatment targeting PSCs is an important focus for TME modulation in PC and has great potential on overcoming chemoresistance.

Tumor associated macrophages (TAMs) are macrophages infiltrating TME and constitute an important part of immune cells. Macrophages are mainly divided into anti-tumor M1 phenotype and protumor M2 phenotype. TAMs mainly keep the function of M2 phenotype (Hao et al., 2021), which is closely related to chemoresistance (Li et al., 2022). HK2 is highly expressed in PC TAMs (Chang et al., 2022), TAMs can also promote Warburg effect through CCL18/NF kB/VCAM-1 pathway (Yang et al., 2020). Besides, TAMs promote aerobic glycolysis of adjacent PC cells through paracrine signals (Qin et al., 2020). Lactate in TME facilitates the polarization of protumor M2 subtype of TAMs, thus forming a positive feedback loop (Ye et al., 2018). In PC xenograft mice models, GEM stimulates TAMs to appear more M2 phenotypes, which increase stroma glycolysis and reduce OXPHOS. Moreover, TAMs upregulate cytidine deaminase, the metabolism enzyme of GEM, and release pyrimidine species (including deoxycytidine), inhibiting the uptake of GEM through molecular competition, both of them lead to GEM resistance (Bulle et al., 2020). Chitinase 3-like-1 and fibronectin in extracellular vesicles released by macrophages reduce the sensitivity of PC cells to gemcitabine, and inhibition of the above two proteins can partially reverse gemcitabine resistance (Xavier et al., 2021). Colony-stimulating factor 1(CSF1), C-C motif chemokine ligand 2 (CCL2) and C-C motif chemokine ligand 2 (CCR2) are essential for the differentiation and chemotaxis of macrophages. Blocking CCL2 with neutralizing antibodies prevent macrophage recruitment and restore the sensitivity of mice PC model to FOLFIRINOX (Nywening et al., 2018)。Inhibition of TAMs by CSF1-receptor antagonist enhances the drug effect of GEM-resistant PC mice models. In the clinical trials of FOLFIRINOX with CCR2 antagonists PF-04136309 and CCX872, respectively, better local tumor control rate was observed (NCT01413022), and the overall survival rate was also improved compared with chemotherapy alone (NCT02345408) (Poh and Ernst, 2021).

### 2.2 OXPHOS and ROS

The mitochondria are important energy factories for ATP production through OXPHOS and are integrated into intracellular signaling networks regulating various cellular functions, making it the hub of tumor cell metabolism. The TCA cycle occurs in mitochondria, producing reducing equivalents of NADH and FADH2 using substrates from the catabolism of three main nutrients. It powers the electron transport chain composed of protease complexes in the inner mitochondrial membrane. Conversely, the mitochondria provide raw materials for the synthesis of biomolecules required for the rapid proliferation of tumor cells, such as amino acids, lipids, and nucleotides. Besides, the mitochondria regulate cellular redox status and ROS production, and mediate endogenous apoptosis. Continuous remodeling of the mitochondria through fusion and division also influences cellular functions. Generally, chemotherapy causes mitochondrial dysfunction and oxidative stress to produce cytotoxic effects for therapeutic purposes. Based on the functions of the mitochondria mentioned above, changes in mitochondrial structure and function are also important factors in chemotherapy resistance.

Metabolic reprogramming of PC cells is accompanied by alterations in redox metabolism and ROS levels. ROS is mostly derived from OXPHOS carried out by the mitochondrial respiratory chain and is a by-product of the electron transport process. Moreover, the electron transfer process of membrane-bound NADPH oxidases can generate ROS (Kalyanaraman et al., 2018). ROS is regulated by the PC common mutant genes KRAS and P53 and can be eliminated by antioxidants. The influence of ROS on cancer cell growth is complicated and correlates with intracellular concentration. Low to moderate levels of ROS induce activation of the PI3K/Akt pathway and promote tumor signaling by interfering with PTEN, protein tyrosine phosphatase 1B, and protein phosphatase 2 (Koundouros and Poulogiannis, 2018). However, excessive ROS concentration imbalances oxidation and antioxidation, causing oxidative stress. The oxidative damage to proteins, nucleic acids and other biomolecules disrupts normal cell structure and functions, leading to cell death. Thus, targeting redox metabolism is a flexible access to antitumor therapy. Nevertheless, studies in recent years show that cancer cells can gradually adapt to the ROS overload environment by increasing antioxidant capacity and activating NF-κB and nuclear factor E2-related factor 2 (NRF2), the master regulator of antioxidant response, to induce tumor progression and chemoresistance. In addition, it has been recently shown that PC cells can acquire a hybrid glycolytic/OXPHOS phenotype. The ATP come from a combination of glycolysis and OXPHOS. In particular, when glycolysis is suppressed, cells can switch from glycolytic phenotype to OXPHOS to meet energy needs (Peng et al., 2021). This suggests that the effect of drugs targeting glycolytic or OXPHOS pathways alone may be limited, and a combination regimen of multiple metabolic inhibitors should be considered.

The mitochondrial genome is divided into two parts: mitochondrial DNA (mtDNA) and nuclear DNA (nDNA). mtDNA encodes 13 proteins in humans and constitutes the core constituent of OXPHOS. Most of the remaining mitochondrial proteins are encoded by nDNA and imported into the mitochondria. Most cancer cells are accompanied by mtDNA mutations and/or mtDNA copy number variations. Cancer cells lacking mtDNA tend to be more sensitive to chemotherapy (Guerra et al., 2017). The transfer of mtDNA cybrids from PC cells to normal cells leads to resistance of progeny cells to 5-FU and cisplatin (Hua et al., 2021). The mtDNA transcriptional inhibitors Mito-Chlor and SQD1 both increase cellular and mitochondrial ROS and inhibit PC cell proliferation (Chen et al., 2020). Furthermore, the resistance of PC cells BxPC-3 to GEM is dependent on mitochondrial-mediated apoptosis *via* the ERK1/2-Bax/Bcl2 signaling pathway (Wang et al., 2015). In Wnt ligand-dependent PC, suppression of porcupine, a Wnt pathway protein, disrupts mitochondrial function and homeostasis and inhibits cancer cell growth in combination with chloroquine (Aguilera et al., 2022). Pyrvinium pamoate, a Wnt inhibitor, also suppresses the mitochondrial pathway, reduces phosphorylation, downregulates mitochondrial RNA transcription, and effectively inhibits the growth of organoids and transplanted tumors (Schultz et al., 2021). Blocking mitochondria-encoded protein synthesis also increases the antitumor effect of GEM (Dijk et al., 2020). The sesquiterpenoid Nardoguaianone L (G-6) improves GEM efficacy by inducing ROS and decreases mitochondrial membrane potential through the AGE-RAGE signaling pathway, leading to apoptosis (Zheng et al., 2022). Laminarin extracted from brown algae also shows synergistic effects in combination with 5-FU by causing mitochondrial damage (Lee et al., 2022).

Mitophagy is a conserved cellular process that effectively deletes dysfunctional mitochondria and maintains intracellular homeostasis. Mitophagy-related genes could be used as predictors for prognostic stratification of PC patients. Patients with high expression of three genes, PRKN, SRC, and VDAC1, are more sensitive to paclitaxel and erlotinib (Zhuo et al., 2021). Mitophagy in PC cells rapidly clears damaged mitochondria to avoid chemotherapy-induced mitochondrial dysfunction and oxidative stress, while limiting glucose flow to mitochondria and driving glycolysis (Humpton et al., 2019). However, excessive mitophagy could lead to loss of functional mitochondria and disrupt cellular energy supply (Wang Y. et al., 2020). OXPHOS can be reduced by mitochondrial fusion through inhibition of dynamin-related protein-1 or overexpression of mitochondrial fusion protein 2, which enhances mitophagy and inhibits PC growth (Yu et al., 2019b). The products of macroautophagy also fuel biosynthesis and power supply, which are also required to maintain mitochondrial function. The RAS oncogenic mutations commonly found in PC could cause cancer cells to rely on autophagy. mtDNA stress also activates autophagy-dependent ferroptosis (Li et al., 2021). In turn, autophagy supports cancer survival through the mitochondrial function and promotes chemoresistance (Reyes-Castellanos et al., 2022).

### 2.3 Pentose phosphate pathway and hexosamine biosynthetic pathway

PPP forms a bypass from the intermediate of glycolysis, glucose-6-phosphate, and returns to the glycolytic pathway through two stages of oxidation and group transfer to generate F-6-P and glyceraldehyde-3-phosphate. PPP cannot produce ATP, but its main purpose is to generate NADPH and ribose phosphate, participating in redox regulation and lipid and nucleic acid production. PPP is divided into oxidative and non-oxidative arms; oxidative PPP (oxPPP) produces NADPH, and non-oxidative PPP (non-oxPPP) produces nucleic acids.

In PC cells, oncogenic KRAS preferentially runs non-oxPPP fluxes to promote DNA/RNA synthesis by upregulating the transcriptional levels of key enzymes RPE and RPIA. Increased glucose flux into non-oxPPP enhances the *de novo* synthesis of pyrimidine derivatives, including dCTP, which competitively diminishes GEM efficacy and causes drug resistance (Yamamoto et al., 2022). Histone H3 lysine 36 trimethylation (H3K36me3) on the promoter of transketolase (TKT) in PPP is indispensable for its transcriptional activation. SET-domain containing 2 (SETD2) is the primary methyltransferase catalyzing H3K36me3. SETD2-deficient PC downregulates TKT transcript levels in PPP to impair nucleoside synthesis while upregulating GLUT-1 to enhance glycolytic addiction, increasing the sensitivity of SETD2-deficient PC cells to GEM when glycolysis is restricted. This suggests that inhibition of glycolysis combined with GEM may be a potential therapeutic strategy for the treatment of SETD2-deficient PC patients (Shen et al., 2022). Increased expression of the calcium-binding protein S100A11 in PDAC promotes the loading of H3K4me3 onto the TKT promoter, enhancing the PPP by increasing TKT levels and leading to worse clinical prognosis and disease progression. This suggests that S100A11 may be a possible therapeutic target (Zeng et al., 2022). PPP is a branch of glycolysis. Substrates of PPP are also influenced by the initial glucose uptake flux during glycolysis. Therefore, inhibitors of GLUT also interfere with the PPP process. For example, Glutipyran, a small molecule broad-spectrum GLUT inhibitor, significantly inhibited the glycolytic pathway and most metabolites of the PPP in PC. It inhibits the growth of PC cells and xenograft mice tumors. When co-treated with the mitochondrial respiration inhibitor metformin, Glutipyran showed synergistic antiproliferative effects (Kawatani et al., 2021).

The Warburg effect predisposes tumors to chronic acidosis, in which PPP is significantly enhanced and promotes proliferation by activating the Yes1 associated transcriptional regulator (YAP)/matrix metalloproteinase-1 axis (Chen S. et al., 2022). PPP is also a mediator for the functions of several tumor regulators. Prolactin receptor short isoform suppresses PPP and nucleotide synthesis *via* the NEK9-Hippo axis, thereby inhibiting cell proliferation in human PC and tumor growth in KPC mice (Nie et al., 2021). Activation of the neuromodulator 5-hydroxytryptamine increases glycolytic flux in PC cells and markedly upregulates metabolic enzymes of glycolysis, PPP, and HBP. Thus, it further promotes the Warburg effect and the growth xenografts PC tumors in mice (Jiang et al., 2017).

PPP functions in nucleotide and lipid synthesis and redox metabolism in PC cells. Excessive activation of PPP tends to promote cancer cell proliferation and chemoresistance. Targeted treatment of this pathway may enable to improve the antitumor effect of chemotherapy.

HBP is another branch of glycolysis responsible for the production of UDP-GlcNAc, a critical substrate for protein glycosylation, using glucose, acetyl coenzyme A (CoA), glutamine (Gln), and UTP. Then, the O-linked β-N-acetylglucosamine (O-GlcNAc) glycosylation modifications completes and regulates cellular functions. Tumor development and drug resistance are often accompanied by abnormal protein glycosylation, both pointing to alterations in HBP.

The HBP activity is enhanced in PC patients and is involved in cell signaling and metabolic reprogramming *via* O-GlcNAcylation. Glutamine-fructose amidotransferase 1 (GFAT1), a key enzyme of HBP, is commonly overexpressed in PC tissues and is associated with poor prognosis in patients with resectable PC (Gong et al., 2021). Silencing GFAT1 reduces O-GlcNAc levels and inhibits PC cell growth (Jia et al., 2020; Yamamoto et al., 2022). Both CD133+ or CD44^+^ PC stem cells have higher GFAT1 levels and are more resistant to GEM-induced apoptosis than cells with corresponding marker loss (Wang Z. et al., 2022). Stomatin like protein 2 is a mitochondrial-associated protein upregulated after GEM stimulation. It activates HBP by upregulating the expression of GFAT2. Although inhibition of SLP-2 does not alter GEM sensitivity, increased expression of SLP-2 contributes to PC liver metastasis (Chao et al., 2021). Phosphoacetylglucosamine Mutase 3 (PGM3) expression in HBP is specifically enhanced in human PC tissues and is associated with poor OS. Suppressing PGM3 reduces protein glycosylation, causes a sustained unfolded protein response, downregulates the pro-tumor EGFR-Akt axis, and ultimately leads to cell death. The combination of PGM3 inhibitor FR054 and GEM increases chemosensitivity and effectively inhibits xenograft tumor growth (Ricciardiello et al., 2020). A recent study showed that hyaluronic acid in PC bypass GFAT1 and promote PC cell growth by replenishing energy for HBP through the GlcNAc salvage pathway (Kim et al., 2021).

The products of various metabolisms (including glucose, lipids, amino acids, and nucleotide metabolism) in PC are pooled in HBP. HBP also coordinates with metabolic pathways and integrate nutrient signals in response to environmental changes. Malate dehydrogenase 1 (MDH1) is a key enzyme in the non-canonical metabolic pathway of Gln in PC and is positively regulated by protein glycosylation. O-GlcNAcylation deficiency decreases MDH1 activity, impairs Gln metabolism, sensitizes PC cells to oxidative stress, and finally inhibits tumor growth *in vitro* and *in vivo* (Zhu et al., 2022). O-GlcNAcylation increases the stability of c-MYC, which promotes the expression of genes related to glycolysis and Gln catabolism, providing energy to tumor cells. Besides, O-GlcNAcylation of the oncogenic factor FOXO3 disrupts its function and leads to PC cell growth (Shin et al., 2018). In addition, O-GlcNAcylation inhibits PFK1 activity and allows glucose to enter PPP, thereby increasing nucleotide and NADPH production for PC cells to adapt to metabolic alterations in the microenvironment. Moreover, glycolysis inhibitor 2-DG can inhibit endoplasmic reticulum stress-mediated PC cell growth by interfering with protein glycosylation and increasing phosphorylation of GFAT1 (Ishino et al., 2018). After receiving extracellular glucose signals, HBP directly regulates the Hippo pathway and drives its core component of YAP O-GlcNAcylation, promoting tumorigenesis (Peng et al., 2017).

## 3 Lipid metabolism

As one of the major nutrients, lipids perform adaptive changes in tumor development. Lipids provide sufficient biofilm components and bioactive substance precursors, serve in energy supply, and are involved in cellular signaling pathways. Key enzymes and regulators involved in lipid metabolism have been proven to be involved in the onset of chemoresistance and may serve as new targets to improve chemosensitivity.

### 3.1 Fat metabolism

Lipids can be biosynthesized or directly absorbed by the diet. Unlike normal cells that are dependent on the diet, approximately 93% of lipids in tumor cells are synthesized from mitochondrial citrate (novo lipid synthesis) (Menendez and Lupu, 2007), also called *De Novo* Lipogenesis (DNL). Reliance on DNL is a remarkable feature of PC cells and one of the key drivers of tumor development and drug resistance.

PC cells are very dependent on DNL, and the levels of various lipogenic enzymes regulated by KRAS are elevated in DNL, such as ATP-citrate lyase (ACLY), FASN, and acetyl CoA carboxylase (ACC) (Swierczynski et al., 2014; Daemen et al., 2015). The silencing of relevant genes, especially *FASN* and *ACC*, significantly inhibits tumor development by limiting lipogenesis. It is also demonstrated that increased levels of lipogenic enzymes are markers of poor prognosis and predict low response to chemotherapy (Sunami et al., 2017). The PI3K/AKT pathway is affected by KRAS, and its downstream molecule sterol regulatory element-binding protein 1 (SREBP1) is an important transcription factor participating in PC growth and regulates lipogenic enzyme expression (Bian et al., 2015). Overexpression of SREBP1 is related to shorter OS of PC patients (Sun et al., 2015). Inhibition of SREBP-1 downregulates FASN and ACC levels, thereby reducing palmitic acid and stearic acid synthesis and notably inhibiting cell proliferation in the BxPC-3 transplantation tumor model (Kim et al., 2022). A recent study showed that KRAS also promotes fatty acid synthesis and energy metabolism by upregulating the expression of phospholipase A2 group IIA, which may be a new therapeutic target as a KRAS downstream molecule (Zhang et al., 2022). Targeting pyruvate dehydrogenase kinase 4 in glucose metabolism also interferes with lipid metabolism and promotes ROS production, thereby reducing the proliferation of KRAS mutation-driven PC cells (Terado et al., 2022). In addition, CDK4/6 inhibitors target miR-33a to regulate AMPK/mTOR signaling and downregulate fatty acid anabolism (Rencuzogulları et al., 2022).

In PC lipid metabolism, the expression level of FASN is critical in shaping chemotherapy resistance. FASN levels are increased in both the tumor tissues and serum of PC patients and are associated with poor prognosis accompanied by low response to GEM (Alo et al., 2007; Fazli et al., 2021). The PI3K/AKT axis and MAPK pathway negatively modulate lipid synthesis by inhibiting SREBP1c levels and thus reducing FASN transcription. Knockout or overexpression of FASN gene significantly downregulates or upregulates the resistance of PC cells to GEM (Yang et al., 2011), in which the acidic tumor microenvironment also plays a pro-drug resistance role (Zuzčák and Trnka, 2022). In PC cells *in vitro* culture and mice transplantation models, the FASN inhibitor orlistat in combination with GEM induced endoplasmic reticulum stress, prompting apoptosis, and markedly reduced PC cell stemness. Direct induction of endoplasmic reticulum stress with thapsigargin (toxic carotene) also caused a similar decrease in stemness and showed a synergistic activity with GEM. This indicates that regulation of enzymes in fatty acid synthesis assists in overcoming GEM resistance in PC by modulating endoplasmic reticulum stress and tumor cell stemness (Tadros et al., 2017). FASN overexpression also upregulates PKM2 expression at the mRNA and protein levels, increasing glycolysis and inducing resistance to GEM (Tian et al., 2018).

TOFA, the ACC inhibitor, enhanced the suppression of PC cell proliferation in a glutamine deprivation environment created by glutaminase (GLS) inhibitor BPTES (Nishi et al., 2018). A novel paclitaxel nanoformulation was recently reported to improve the efficacy of GEM in PC by decreasing the expression of FASN and ACC, preventing DNL and disrupting membrane stability (Shetty et al., 2020).

Stearyl CoA desaturase 1 (SCD1) expression is elevated in PC, promotes the synthesis of unsaturated fatty acids (FAs), and is controlled by SREBP1. The yarrow supercritical extract inhibits tumor growth in PC transplantation mice models by downmodulating SREBF1 and its downstream targets, SCD and FASN (Mouhid et al., 2019). SCD1 repression also interfered with aberrant RAS and AKT signaling pathways in PC, suggesting that SCD1 may be a potential therapeutic target in lipid metabolism (Eser et al., 2014).

It is currently proposed that in PC, saturated, monounsaturated, and ω-6 polyunsaturated FAs have pro-cancer cell growth effects (Qin et al., 2020). ω-3 polyunsaturated FAs, on the other hand, improve the anti-cancer effects of GEM by reducing AKT phosphorylation and suppressing NF-kβ and STAT3 activation, thereby arresting cancer cell proliferation (Ding et al., 2018). DHA and EPA are both ω-3 polyunsaturated FAs, and their co-agent Lipidem™, combined with GEM, suppresses the growth of PC cells and PSCs, with PSCs being particularly sensitive (Haqq et al., 2016). Arachidonic acid (AA) is an ω-6 polyunsaturated FA, and leukotrienes are one of the metabolites produced by AA in response to lipoxygenase (LOX). Aberrant metabolism of AA *via* the LOX pathway is a common feature of epithelial-derived malignancies, including PC. LOX genes are upregulated in multiple cancers, particularly 5-LOX, which enhances the proliferation rate and invasive capacity of tumor cells and induce stronger anti-apoptotic signals (Mahboubi-Rabbani and Zarghi, 2021). Both leukotriene receptor antagonists and 5-LOX inhibitors are already in clinical trials of several diseases (Vishnupriya et al., 2021).

Current studies have confirmed the importance of fat synthesis in PC chemoresistance, that reducing adipogenic signaling could help solve the problem. However, FAs released *via* food intake and fat tissues, although representing only a very small portion of the FAs source in PC cells, may still be a hindrance to the effect of inhibitors targeting FA synthesis. Therefore, multiple metabolic inhibitors in combination or with chemotherapy may be considered to meet the practical clinical needs.

### 3.2 Cholesterol metabolism

Cholesterol is an essential structural component of cell membranes, in the form of free cholesterol and cholesteryl esters, which support the proliferation and migration of cancer cells (Chimento et al., 2018). Acetyl CoA is first converted to 3-hydroxy-3-methylglutaryl-coenzyme A (HMG-CoA) through a series of steps, and then reduced by HMG-CoA reductase (HMGCR) to mevalonate (MVA), then converted by some metabolic steps to farnesyl pyrophosphate (FPP). Finally, by the action of the acyl-coenzyme A cholesterol acyltransferase (ACAT), intracellular free cholesterol is condensed with acyl-coenzyme A to form cholesteryl esters for storage. Low-density lipoprotein (LDL) mainly transports endogenous cholesterol. Plasma LDL is endocytosed into cells after binding to LDL receptors (LDLR), and cholesteryl esters are hydrolyzed into free cholesterol and fatty acids.

HMGCR is a key enzyme in cholesterol synthesis and is markedly overexpressed in PC (Guillaumond et al., 2015). The cholesterol synthesis products MVA and free cholesterol are allosteric inhibitors of HMGCR. Aldo-keto reductase family 1B10 (AKR1B10) is highly expressed in PC patient samples. Its reduction products, FPP and geranylgeranyl pyrophosphate (GGPP), are intermediates in cholesterol synthesis and assist in promoting cholesterol synthesis. FPP and GGPP are also involved in the isoprenoid modification of KRAS proteins, the most common of PC gene mutations, activating downstream signaling molecules (including ERK, MEK, E-cadherin) and promoting PC growth (Chung et al., 2012; Zhang et al., 2014). Silencing AKR1B10 increases apoptosis and inhibits the above-mentioned survival signaling pathways, which could be a possible target for antitumor metabolism. The combination of GEM with SNIPER-11, which targets the cholesterol metabolism regulator CRABP-II, destabilizes SREBP-1c. This in turn significantly reduces cholesterol accumulation in lipid rafts and inhibits PDAC xenograft progression, suggesting that CRABP-II is a possible target to overcome chemoresistance (Yu et al., 2022). SREBP2 also regulates cholesterol synthesis. KRAS can increase cholesterol synthesis by upregulating HMGCR through SREBP2 (Muyinda et al., 2021).

Avasimibe, an ACAT-1 inhibitor, in combination with GEM resulted in suppression of AKT signaling and effectively reduced cholesteryl ester accumulation in GEM-resistant PC cells, which also exhibited strong synergistic antitumor effects in culture *in vivo* (Li et al., 2018). Cholesterol and sphingolipids are also essential components of the lipid raft subdomain caveolae, which support endocytosis of NAB-paclitaxel by caveolae. The level of its major constituent, caveolin-1, is positively correlated with NAB-paclitaxel sensitivity. Downregulation of caveolin-1 levels in PC xenograft mice model induces NAB-paclitaxel resistance (Chatterjee et al., 2017).

Compared with the enhancement of cholesterol synthesis pathway, the upregulation of cholesterol uptake has a more dominant role in PC cells. LDLR is highly expressed at all stages of PC and is associated with an increased risk of recurrence. Silencing LDLR with shRNA reduces the cholesterol uptake and proliferation capacity of PC cells, restricts the activation of the ERK1/2 survival pathway, and significantly improves the efficacy of GEM in mice transplanted tumor models. KRAS may also promote cholesterol uptake in cancer cells by upregulating LDLR (Guillaumond et al., 2015). Therefore, LDLR is a metabolic target for combination therapy, which helps to overcome drug resistance and limit the progression of PC.

Statins are inhibitors of cholesterol synthesis, which reduce exogenous cholesterol uptake by suppressing LDLR on the one hand and inhibit the key enzyme HMGCR on the other (Zuzčák and Trnka, 2022). Lovastatin inhibits the MVA pathway to interfere with membrane structural stability and impairs the PC tumor-initiating cell (TIC) marker CD133 localized on lipid rafts. It causes FAK signaling uncontrolled, inhibits ABC transporter protein activity, and reduces the metastatic invasive capacity and chemoresistance of CD133Hi cells (Gupta et al., 2018). This suggests that targeting lipid rafts with statins sensitizes drug-resistant pancreatic TIC to chemotherapy and reduces metastasis. The combination of atorvastatin with tipifarnib (farnesyltransferase inhibitor) and celecoxib (COX-2 inhibitor) strongly depresses the formation of Panc-1 cell spheroids, inhibits the proliferation of GEM-resistant sphere-forming cells, and promotes apoptosis. This effect is related to the reduction of CD44, CD133, and ALDH1A1 levels and the suppression of Akt and NF-κB activation (Xu X. T. et al., 2021). Moreover, statins promote apoptosis in tumors by depleting isoprenoids such as FPP and GGPP and interfering with the prenylation of signaling proteins such as KRAS (Jiang et al., 2021a; Nam et al., 2021).

## 4 Amino acid metabolism

Amino acids are the basic building blocks of proteins and a very valuable metabolic fuel for tumor cells. Unlike normal cells, the reliance of cancer cells on the glucose glycolytic pathway results in a greater conversion of generated pyruvate to lactate, away from the TCA cycle and subsequent fatty acid synthesis link in the mitochondria (Wang Z. et al., 2020). This downregulates the level of acetyl CoA production in mitochondria, which consequently reduces the production of ATP, biosynthetic precursors, and reducing equivalents, making the support from Gln metabolism a critical one. As the most abundant amino acid in human blood circulation, Gln is converted to α-ketoglutarate (α-KG) through metabolism. α-KG enters the TCA cycle as an intermediate product, supports the normal functioning of the TCA cycle through the backfill effect, and serves DNL. Thus, cancer cells are distinctly Gln-dependent for survival, and metabolic rearrangements of amino acids in PC cells are also most marked by alterations in the metabolism of Gln, which is an excellent target for improving chemotherapy sensitivity (Kaira et al., 2012; Coothankandaswamy et al., 2016).

Gln enters cells *via* the amino acid transporter protein alanine/serine/cysteine transporter 2 (ASCT2)/SLC1A5 (Bhutia et al., 2015) and is cleaved by GLS in mitochondria to produce glutamate and free ammonia. Glutamate is deaminated by glutamate dehydrogenase (GDH) or transaminated by alanine transaminase (ALT/GPT) or aspartate transaminase (AST/GOT) and converted to α-KG, a key intermediate in the TCA cycle. A high level of α-KG is also a pro-carcinogenic metabolite (Anderson et al., 2018). HIF-1α is activated in hypoxia, inducing a decrease in PDH and α-KG dehydrogenase (α-KGDH) levels in PC cells and shifting the metabolism to isocitrate dehydrogenase (IDH) preferentially mediated metabolism for fatty acid synthesis (Hao et al., 2021). In the cytoplasm, Gln contributes its γ-nitrogen to nucleotide synthesis and also produces reduced glutathione (GSH), which has a very critical role in maintaining redox homeostasis and resistance to ROS oxidative damage (Ju et al., 2020). After entering the cell *via* SLC1A5, Gln is exchanged for other amino acids *via* the reverse L-type amino acid transporter 1/SLC7A5. The export of Gln outside cell in exchange for essential amino acids (e.g., leucine) inside the cell could activate the mTORC1 signaling pathway and promote tumor growth (Nicklin et al., 2009).

Unlike the classical Gln mitochondrial metabolism, Gln is converted into glutamate, then to α-KG, then goes into the TCA cycle in most cells. PC strongly relies on the non-canonical metabolism of Gln to maintain redox homeostasis and cell proliferation. That is, Gln is converted to glutamate in the mitochondria by GLS, catalyzed by GOT2 to aspartate, transferred from the mitochondria by the uncoupling protein 2, converted to oxaloacetate in the cytoplasm by GOT1, converted to malate, then turned to pyruvate, and released as NADPH by malic enzyme (ME) and MDH1 to maintain redox homeostasis. Gln deprivation or genetic inhibition of any enzyme in this pathway leads to an increase in ROS and a decrease in reduced GSH. Knockout of any enzyme or transporter in this pathway (e.g., GLS, MDH, UCP2) significantly inhibits PC cells growth *in vitro* and *in vivo* (Son et al., 2013; Wang et al., 2016; Raho et al., 2020).

This reprogramming of Gln metabolism is mainly mediated by the signature KRAS mutation in PC. Gln metabolism is converted into a non-canonical pathway for NADPH production by regulating the levels of key enzymes, in particular increasing GOT1 and inhibiting GDH1 (Pupo et al., 2019). In hypoxia, GOT1 expression can be promoted by targeting HIF-2α *via* PI3K/mTORC2 (Li et al., 2017). In addition, proto-oncogene c-MYC promotes Gln intake and catabolism through activation of SLC1A5, SLC7A5, and GLS (Wise et al., 2008). Heterozygous deletion of KrasG12D can also promote the malignant phenotype of PC through activation of HIF-2α-c-MYC-regulated Gln metabolism (Ma et al., 2022). Activation of the lnc RNA XLOC_006390/c-MYC also increases GDH1 mRNA expression and promotes glutamate metabolism, which is associated with poor survival in PC patients and is a potential therapeutic target for PC (He et al., 2020). P53 could play a pro-survival role during Gln deprivation, increasing arginine uptake by stimulating SLC7A3 expression, which in turn activates mTORC1 to promote tumor growth (Lowman et al., 2019). The common deletion of Smad4 with ME in PC reduces the production of NADPH and the transcription of branched-chain amino acid transferase 2 (BCAT2) *via* the AMPK/SREBP1 pathway. It inhibits amino acid transfer from branched-chain amino acids to α-KG, and ultimately impacts both glutamate and nucleotide synthesis. Upregulation of ME and NADPH may be potentially beneficial for PC patients with Smad4 homozygous deletion (Dey et al., 2017).

Chemoresistance in PC cells is enhanced or suppressed when Gln metabolism is overexpressed or inhibited, respectively. Suppression of Gln metabolism weakens EGFR signaling and the downstream AKT-mTOR pathway, the MAPK pathway. It affects cellular redox homeostasis, glucose synthesis of HBP, and other metabolic processes, which ultimately leads to proteomic alterations in cancer cells and exosomes and significantly increases chemotherapy sensitivity of cancer cells (Chen et al., 2017). KRAS induces drug resistance by promoting NRF2 expression and enhancing Gln metabolism. GLS inhibitors sensitize GEM-resistant PC cells (Mukhopadhyay et al., 2020). L-type amino acid transporter 2, an oncogenic protein in PC cells, Gln-dependently activates mTOR to inhibit apoptosis and promote glycolysis and GEM resistance. mTOR inhibitor (RAD001) reverses this resistance (Qin et al., 2020). The mucin 5AC (MUC5AC)/β-catenin/c-MYC axis promotes Gln uptake and utilization by PC cells. Thus, targeting this axis increases GEM efficacy (Ganguly et al., 2022).

GOT1 is a key enzyme highly expressed in non-canonical metabolism of Gln. A high ratio of serum AST (GOT)/ALT before NAB-paclitaxel and GEM chemotherapy in advanced PC patients is associated with poor prognosis and can be used as a new indicator for risk assessment of individualized treatment of PC (Riedl et al., 2020). GOT1 inhibitors Aspulvinone O, Ziprasidone, and Aspulvinone H induced the disturbance of Gln metabolism and imbalance of redox status in PC cells, thereby inhibiting cancer cell proliferation, and showed significant antitumor effects in xenograft tumor models (Sun et al., 2019; Yang et al., 2022) (Yan et al., 2021). Suppression of GOT1 also promotes PC cell death by triggering ferroptosis (Kremer et al., 2021). SIRT5, a tumor suppressor of PC, reduces non-canonical metabolism of Gln by deacetylating GOT1 to inhibit its activity, which is associated with a favorable prognosis (Kumar and Lombard, 2021). The selective SIRT5 activator MC3138 has antitumor effects on PC cells and increases the sensitivity of cancer cells and organoids to GEM by reducing the nucleotide pool (Hu et al., 2021). miR-433-3p and miR-9-5p also regulate GOT1 expression and inhibit PC cell proliferation (Wang J. et al., 2019; Zhou et al., 2021).

ASCT2/SLC1A5 is highly expressed in PC and is a primary transporter of Gln. Knockout of ASCT2 induces apoptosis and represses transplantation tumor growth *via* the Akt/mTOR signaling pathway (Wang W. et al., 2022). A novel variant of the SLC1A5 gene induced by HIF-2α enhances GEM resistance in PC cells by inducing GSH synthesis (Yoo et al., 2020). In nutritional deficiency, the activated RNA-binding protein HuR upregulates IDH1 levels, maintains redox homeostasis, and leads to chemoresistance (Zarei et al., 2017). Dicer is high in PC and positively correlates with acquired GEM resistance. This may occur through upregulation of GLS/glutamine synthetase ratio, which affects miRNA maturation and Gln metabolism (Park et al., 2022). Recently, a nanomaterial-based siKRAS/siGLS1 joint reagents was also reported to inhibit both glucose and Gln metabolism, avoiding mutual replacement of the two major metabolic pathways and effectively inhibiting the growth of transplanted tumors (Xu et al., 2022). This could be used as an adjuvant to chemotherapy.

miR-1291 targets argininosuccinate synthase 1 and GLUT1 in PC cells. It suppresses arginine catabolism and glycolysis and effectively enhances the sensitivity of PC cells to arginine deprivation and cisplatin (Tu et al., 2020). Both polyamine biosynthesis (ornithine decarboxylase) inhibitor DFMO and polyamine transport inhibitor Trimer44NMe produce remarkable proliferation inhibition of GEM-resistant PC cells. The combination also significantly prolongs the survival time of tumor-bearing mice (Gitto et al., 2018). Branched chain amino acids (BCAA) and BCAT2 are both highly expressed in PC and have important roles in cell proliferation. BCAT2 promotes BCAA uptake and catabolism, and the carbon provided by BCAA oxidation can be processed into acetyl CoA, which enters the TCA cycle and serves for lipid synthesis. Knockdown of either *BCAT2* gene or branched-chain α-keto acid dehydrogenase a (BCDHA) notably inhibits proliferation and fatty acid production in PC cells. Acetylation also leads to degradation of BCAT2 to inhibit cancer growth (Lei et al., 2020). This suggests that BCAA may be a carbon source for fatty acid biosynthesis in PC cells, and that targeting BCAT2 or reducing dietary BCAA is a therapeutic approach for PC (Lee et al., 2019; Li et al., 2020).

**FIGURE 1:**
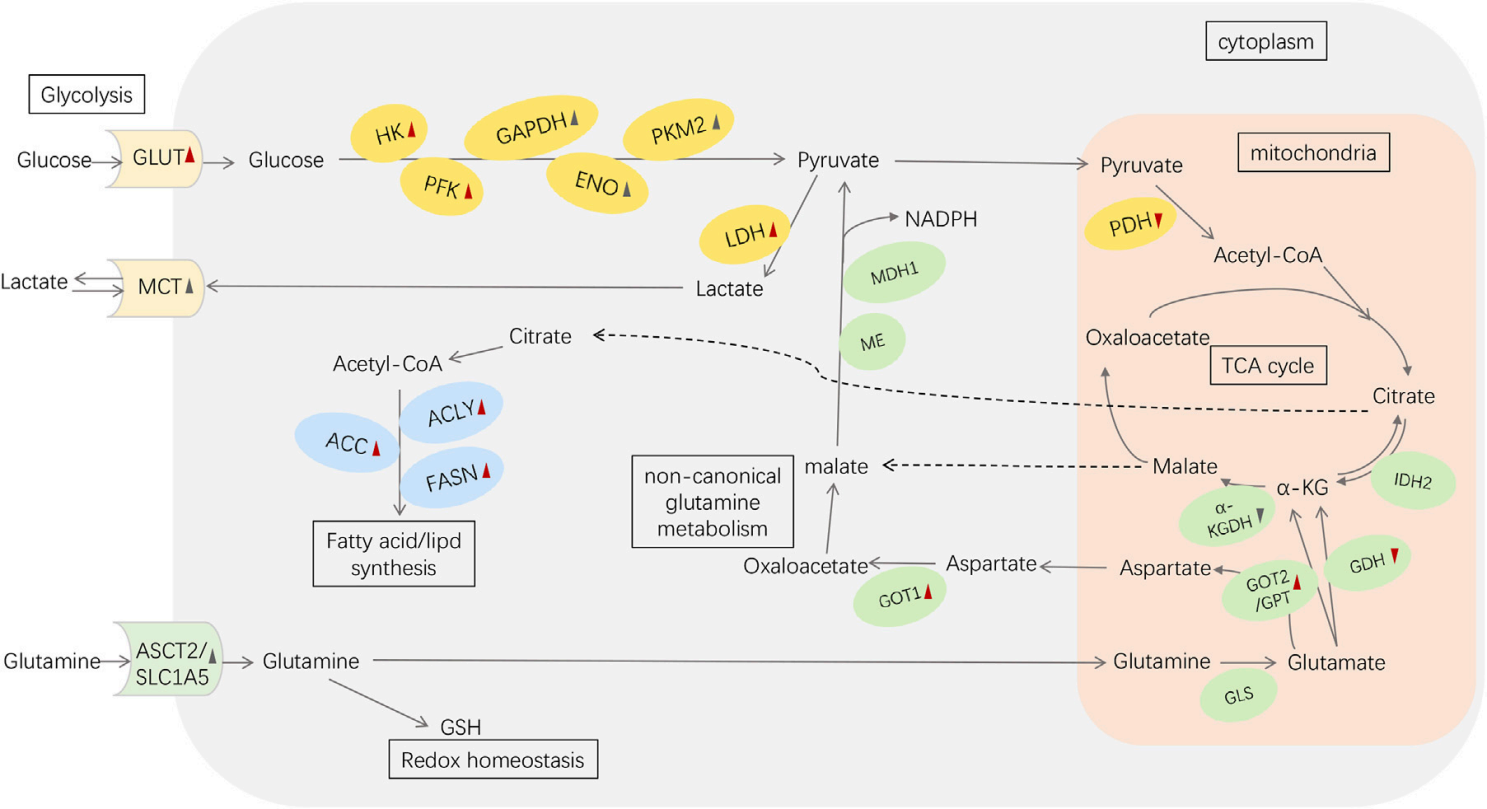
Metabolic reprogramming in pancreatic cancer. Red arrowheads represent the effects induced by mutant KRAS: upward means upregulation; downward means downregulation. The black symbols represent the changes induced by other reasons (e.g., HIF-1α、c-MYC). Abbreviations: GLUT, glucose transporter; HK, hexokinase; PFK, phosphofructokinase; GAPDH, glyceraldehyde-3-phosphate dehydrogenase; ENO, enolase; PK, pyruvate kinase; LDH, lactate dehydrogenase; MCT, monocarboxylic acid transporters; PDH, pyruvate dehydrogenase; α-KG, α-ketoglutarate; ASCT2/SLC1A5, alanine/serine/cysteine transporter 2/SLC1A5; GSH, glutathione; GLS, glutaminase; GDH, glutamate dehydrogenase; IDH2, isocitrate dehydrogenase 2; GOT1, aspartate transaminase 1; GOT2, aspartate transaminase 2; GPT, alanine transaminase; α-KGDH, α-KG dehydrogenase; ME, malic enzyme; MDH1, malate dehydrogenase 1; ACLY, ATP-citrate lyase; ACC, acetyl CoA carboxylase; FASN, fatty acid synthase.

Overall, enzymes and transporters of the various components of PC metabolic reprogramming can be targets for regulating metabolic antitumor therapy (Figure 1).

## 5 Discussion

In the last decade, the metabolic reprogramming of PC has been extensively well studied, proposing the role of the specificity and dependence of metabolism in tumor development and drug resistance. The metabolism of glucose, amino acids, and lipids in PC undergoes adaptive modifications from the cells to the microenvironment, deeply affecting the tumor progression. Studies have shown that targeting different aspects of metabolic rewiring such as aerobic glycolysis, Gln catabolism, and DNL have synergistic effects with chemotherapeutic agents and can significantly improve chemosensitivity in PC (Table 1), giving a pavement for clinical translation. In particular, the treatment of glycolytic pathways is gaining wide attention. In phase I clinical trials in patients with advanced pancreatic cancer, 2-DG in combination with docetaxel has shown actual clinical benefits (NCT00096707) (Qin et al., 2020). In phase I clinical trials of metastatic pancreatic cancer, the combination of CPI-613 and modified FOLFIRINOX had a slight increase in side effects and toxicity, but the response rate was satisfactory (NCT01835041) (Alistar et al., 2017). Although most inhibitors are still in the preclinical stage, glycolysis inhibition represents a promising approach to PC therapy.

**TABLE 1:** Inhibitors used in combination with chemotherapy to overcome drug resistance.

Targets	Inhibitors	Introductions
GLUT	NHI-Glc-2	Targets LDHA and blocks glycolysis El Hassouni et al. (2020)
	miR-1291	Targets GLUT1 to inhibit arginine catabolism and glycolysis Xu et al. (2022)
SGLT	CanA	Downregulates GLUT1 and LDHA levels and hinders glycolysis Xu D et al. (2020)
HK	2-DG	Glucose analogues, cannot be further metabolized after being phosphorylated and accumulate intracellularly to impede glycolysis Dai et al. (2020)
	IKA	Binds to HK2, inhibits its activity and hinders glycolysis Jiang et al. (2020)
PFK	EGCG	Inhibits extracellular acidification and hinders glycolysis Wei et al. (2019)
PK	EGCG	Inhibits extracellular acidification and hinders glycolysis Wei et al. (2019)
	PTBP1	Upregulated PTBP1 level promotes PKM2 isoform production and enhances glycolysis Calabretta et al. (2016)
LDH	Fam83D	Mitosis-related genes, knockdown reduces c-MYC and LDHA expression *via* Wnt/β-catenin signaling pathway Hua et al. (2021)
	NHI	Regulate E2F1 to increase dCK expression required for GEM metabolism, increases GEM-containing DNA synthesis, and affects cell cycle-induced apoptosis Maftouh et al. (2014)
	NHI-Glc-2	Targets LDHA and blocks glycolysis El Hassouni et al. (2020)
MCT	7ACC2	Reduces reverse Warburg effect in MCT1-positive PC cells Sandforth et al. (2020)
HIF-1α	Digoxin	Inhibits downstream aerobic glycolysis-related enzymes and reversals GEM resistance Shukla et al. (2017)
PI3K/Akt	Evr	mTOR inhibitor, promotes apoptosis and inhibits glycolysis Cui et al. (2021)
	RAD001	mTOR inhibitor, promotes apoptosis and inhibits glycolysis Qin et al. (2020)
	Berberine	Regulates Rap1/PI3K/Akt signaling pathway Okuno et al. (2022)
	4-AAQB	Regulate PI3K/Akt/MDR1 signaling pathway Chen Y. Y. et al. (2022)
KRAS	FBP1	Gluconeogenic key enzyme that inhibits GEM-activated ERK by inhibiting the IQGAP1/ERK1/2 signaling pathway Jin et al. (2017)
TME	KI-301690	DDR1 inhibitor, reduces TME matrix components and mitochondrial membrane potential to improve drug delivery efficiency Ko et al. (2022)
	Calcipotriol	Vitamin D receptor ligand on PSC, contributes to PSC restitution and improves PC sensitivity to GEM Sherman et al. (2014)
	ProAgio	Targets integrin α_v_β_3_ highly expressed on PSC, induces PSC apoptosis, reduces vascular compression by ECM, improves drug transport, and reduces the level of GEM metabolic enzyme cytidine deaminase Turaga et al. (2021)
	PF-04136309	CCR2 antagonist, blocks the main chemotactic signal of macrophages, reduces the number of TAMs and hinders tumor growth Nywening et al. (2016)
	CCX872	CCR2 specific antagonist, blocks the main chemotactic signal of macrophages, reduces the number of TAMs and hinders tumor growth Poh and Ernst (2021)
HBP	FR054	Inhibitor of PGM3, reduces protein glycosylation, causes sustained unfolded protein response, downregulates the pro-tumor EGFR-Akt axis, and ultimately leads to cell death Ricciardiello et al. (2020)
DNL	Orlistat	FASN inhibitor, induces endoplasmic reticulum stress, drives apoptosis and reduces PC cell stemness Tadros et al. (2017)
	PPNPs	Reduces the expression of FASN and ACC, inhibits DNL and destabilizes the membrane Shetty et al. (2020)
	Lipidem ™	Co-agent of ω-3 polyunsaturated fatty acids DHA and EPA, reduces AKT phosphorylation, inhibits activation of NF-kβ and STAT3, and suppresses cancer cell proliferation Haqq et al. (2016)
	Avasimibe	ACAT-1 inhibitor, downregulates AKT signaling and reduces cholesteryl ester accumulation in cells Li et al. (2018)
	Lovastatin	Cholesterol synthesis inhibitor, inhibits the MVA pathway interfering with membrane structural stability, impairs the TIC marker CD133 localized on lipid rafts, causes loss of FAK signaling and inhibits ABC transporter protein activity Gupta et al. (2018)
	Atorvastatin with tipifarnib and celecoxib	Cholesterol synthesis inhibitor, farnesyltransferase inhibitor and COX-2 inhibitor, decrease CD44, CD133 and ALDH1A1 levels, inhibit Akt and NF-κB activation, inhibit cancer cell spheroid formation and promote apoptosis Xu X. T. et al. (2021)
Glutamine metabolism	6-diazo-5-oxo-L-norleucine (DON)	Gln analog, interferes with nucleotide and protein synthesis pathways and inhibits Gln metabolism Chen et al. (2017)
	CB-839, BPTES	Inhibitor of GLS, inhibits Gln metabolism Mukhopadhyay et al. (2020)
	CPI	Inhibitors of PDH and α-KGDH, blocks TCA cycle, interferes with cellular energy supply, biomolecule synthesis and redox homeostasis Alistar et al. (2017)
	MC3138	Selective activator of SIRT5, deacetylation of GOT1 to inhibit its activity, reduces non-canonical metabolism of Gln, and suppresses the increase of nucleotide pool Kumar and Lombard (2021)
	miR-1291	Targets ASS1 and inhibits arginine catabolism Tu et al. (2020)
	DFMO	Targets ornithine decarboxylase to inhibit polyamine biosynthesis Gitto et al. (2018)
	Trimer44NMe	Inhibits polyamine transport Gitto et al. (2018)
Mitochondria	Nardoguaianone L (G-6)	Sesquiterpenoid, induces ROS and decreases mitochondrial membrane potential *via* AGE-RAGE signaling pathway, leading to apoptosis Zheng et al. (2022)
	Laminarin	Causes mitochondrial membrane depolarization, calcium ion disruption, and inhibition of signaling pathways related to ROS production Lee et al. (2022)

With the Warburg effect as the starting point, the huge and complex regulatory network behind cancer metabolism is gradually emerging. PC shows a high degree of metabolic plasticity. In addition to changes in its internal key links, its metabolic pathways also coordinate with each other and can interact with characteristic gene changes (such as KRAS, TP53, and c-MYC) in PC. The TME also plays a considerable role. Therefore, inhibition of a certain metabolic pathway or key enzyme may not be enough to block the progress of the whole tumor metabolic rewiring to promote growth and drug resistance. Moreover, the lack of resistance markers and prognostic indicators for inhibitors, as well as the suboptimal specificity of inhibitors, are obstacles to the entry of metabolic inhibitors into clinical practice. In most guidelines for multiple cancers, metabolism-targeted therapy has not yet been recommended as routine treatment. Most therapies targeting PC metabolism are still in preclinical trials.

Although clinical evidence for the use of metabolic inhibitors to overcome PC chemoresistance remains to be improved, the vital role of metabolic reprogramming in impacting chemotherapy sensitivity cannot be denied. While trying to identify the real control switch of metabolic rewiring, we should also start to develop joint reagents that can selectively work on multiple molecular targets. Overall, targeted metabolic reprogramming is currently a reasonable idea and an effective solution to address PC chemotherapy resistance. It is hopeful to see more patients receive metabolic targeted therapy combined with chemotherapy to improve their clinical outcomes in the future.
